# Editorial: Genetic characterization of yield- and quality-related traits in legumes

**DOI:** 10.3389/fpls.2023.1281138

**Published:** 2023-10-17

**Authors:** Zhengjun Xia, Qijian Song, Kyuya Harada, Jianghua Chen, Chuanen Zhou

**Affiliations:** ^1^ Key Laboratory of Soybean Molecular Design Breeding, Northeast Institute of Geography and Agroecology, The Innovative Academy of Seed Design, Chinese Academy of Sciences, Harbin, China; ^2^ United States Department of Agriculture- Agriculture Research Service (USDA ARS), Soybean Genome & Improvement Lab, Beltsville, MD, United States; ^3^ Department of Biotechnology, Graduate School of Engineering, Osaka University, Suita, Japan; ^4^ CAS Key Laboratory of Topical Plant Resources and Sustainable Use, CAS Center for Excellence in Molecular Plant Sciences, Xishuangbanna Tropical Botanical Garden, Chinese Academy of Sciences, Kunming, China; ^5^ The Key Laboratory of Plant Development and Environmental Adaptation Biology, Ministry of Education, School of Life Sciences, Shandong University, Qingdao, China

**Keywords:** legume, yield, quality, soybean, gene cloning, functional characterization, Medicago, plant architecture

Legumes (Fabaceae) are essential crop plants throughout the world, second in importance only to members of the grass family (Poaceae) (Smýkal et al., 2015). One-third of human dietary protein comes from grain legumes, which represent more than one-fourth of global crop production (Smýkal et al., 2015). Legumes are vital for agriculture, natural ecosystems, and agroforestry owing to their capacity for symbiotic nitrogen fixation (Smýkal et al., 2015). The complex nature of traits associated with yield and quality poses challenges for studying their underlying molecular mechanisms. However, gene cloning and functional studies of traits that are unstable and dependent on the environment are being enhanced through various omics technologies. There were a total of 26 submissions for this Research Topic on the genetic characterization of yield- and quality-related traits in legumes, 13 of which were eventually accepted for publication. These papers fall into five subtopics: (1) plant architecture and leaf development, (2) pod and seed traits, (3) seed composition, (4) abiotic stress resistance, and (5) nodulation.

## 1 Plant architecture and leaf development

The plant architecture of members of the legume family starkly contrasts with that in the grass family, Poaceae. Even though it appears that only a few key genes were targeted by selection during plant domestication and breeding, plant architecture can confer optimal plasticity in response to environmental changes. Moreover, plant architecture has considerable effects on the seed yield of grain legumes, a major source of human dietary protein, which is poised to become increasingly crucial as human diets shift toward reduced animal protein consumption for health and environmental reasons. However, there has been limited research on the molecular regulation of plant architecture in legumes to date.

Li X. et al. investigated the roles of MATE/DTX (Multidrug and toxic compound extrusion/detoxification) family transporters in controlling plant development as well as stress responses. The authors identified a *mini body 1* (*mib1*) mutant in mung bean (*Vigna radiata*) that shows increased branching, five-leaflet compound leaves, and reduced pod length. Through map-based cloning, they found that *MIB1* encodes a MATE/DTX family protein in mung bean. Despite expression in all tissues, quantitative reverse transcription PCR (qRT-PCR) results demonstrated that young inflorescences exhibit the highest expression of this gene. A complementation assay in *Escherichia coli* suggested that MIB1 functions as a MATE/DTX transporter, while the short-pod phenotype of the Arabidopsis (*Arabidopsis thaliana*) *dtx54* mutant could be partially rescued by heterologous expression of mung bean *MIB1*. Transcriptome analysis indicated that the molecular function of MIB1 might involve a phytohormone pathway. Taking their results together, the authors concluded that MIB1 is important for controlling the establishment of plant architecture in mung bean.

Meanwhile, in a study of the AINTEGUMENTA-LIKE (AIL) family in *Medicago truncatula*, a model legume for functional studies of leaf development, Wang et al. revealed that AINTEGUMENTA (ANT) proteins have important roles in leaf growth. Four *MtANT* genes were present among the 11 *MtAIL* genes they identified. High expression levels of *MtANT1* were observed in tissue containing active stem cells, e.g., the shoot apical meristem and leaf primordium. Further study of *MtANT* quadruple mutants as well as plants overexpressing *MtANT* revealed that MtANTs control leaf size by regulating cell proliferation during secondary morphogenesis in *M. truncatula* leaves. This study is the first to reveal the function of MtANTs during leaf growth. Further work will help to elucidate the molecular mechanism whereby MtANTs regulate leaf size. Because biomass is critical for imrpoving forage grass quality, genetic manipulation of these genes could enhance the biomass production of legume forage crops.

## 2 Pod and seed traits

Seed size and shape are important quality traits of lentil (*Lens culinaris* Medik.), affecting the yield of milled grain, market grade, and cooking time. Pod traits are important components of yield in snap bean (*Phaseolus vulgaris*) and other fresh legume crops. There are rich genetic resources for seed and pod traits such as size and color among different leguminous species and even within a given species, e.g., common bean. Functional studies of the regulatory networks in different leguminous species will shed light on the molecular bases of seed and pod traits.

Luo et al. focused on quantitative trait locus (QTL) mapping and gene mining for two closely correlated seed traits, seed size and seed weight, in soybean (*Glycine max*) grown in different environments. They identified 18 environmentally stable QTLs among 85 QTLs associated with seed size and weight, which they mapped using a recombinant inbred line (RIL) population developed from Guizao1×B13 (GB13). Notably, the newly identified QTL qSL-3-1 showed a stable effect, contributing to 10.0% to 15.91% of phenotypic variance (PV) in seed length. In addition, qSW20-3 had a stable effect on seed width, explaining 9.22% to 21.93% of PV. Functional annotation and GO enrichment analysis identified 15 candidate genes with possible roles in controlling seed size and weight in soybean. These results provide a reference for studying the development of soybean seed, which will influence molecular breeding and enable consistent enhancement of soybean yield.

The proportion of four-seeded pods (PoFSP) is an important factor contributing to soybean yield. The *Ln* gene was previously shown to pleiotropically control leaflet shape and seed number in soybean. Cao et al. performed fine mapping and candidate gene analysis of the PoFSP in soybean using a chromosome segment substitution line (CSSL) population. Eleven QTLs were identified in the CSSL population, with all 14 genes annotated in the delimited QTL intervals showing variation in the promoter region or coding sequence. Five candidate genes were differentially expressed in pooled accessions displaying opposing phenotypic extremes (PoFSP >35.92% for high pool; PoFSP <17.56% for low pool). Haplotype analysis was consistent with this finding. The results of this work will greatly facilitate the study of candidate genes affecting soybean PoFSP and provide a foundation for marker-assisted selection of the four-seeded pod trait.

Limited information is available on the molecular basis of pod dimension or size in common bean (snap bean). Li M. et al. used genome-wide association analysis to identify the optimal genomic regions for improving pod size in snap bean. Analysis of 88 snap bean accessions they revealed 57 SNPs significantly linked with the pod size trait. Of the 26 candidate genes for pod development, which included cytochrome P450 family genes and WRKY and MYB transcription factor genes, eight showed high expression levels in flowers as well as young pods. These findings increase our understanding of the genetic basis of pod size in snap bean.

Dutta et al. identified genes controlling seed size in lentil through morpho-biochemical characterization of a RIL (F5:6) population derived from a cross between L830 (20.9 g per 1000 seeds) and L4602 (42.13 g per 1000 seeds). A parental polymorphism survey using two extreme phenotype pools and 394 simple sequence repeats (SSRs) identified 31 polymorphic primers. The marker PBALC449 could clearly differentiate the parents from the pool of small seed size. A few candidate genes were identified in the PBALC449 anchored region that might play roles in regulating seed size, including genes encoding ubiquitin C-terminal hydrolase and E3 ubiquitin ligase, as well as other proteins or enzymes such as hexosyltransferase and TIFY-like protein. This study thus improves our understanding of a genomic region controlling seed size in lentil.

## 3 Seed composition

Understanding the molecular mechanisms that regulate seed composition in legumes is important not only for improving seed quality but also for enhancing the nutritional and physiological value of these crops.

Xu et al. investigated the molecular regulation of protein and oil contents in soybean seed via transcriptomic and metabolomic analyses. The authors identified 12,712 differentially expressed genes and 315 differentially accumulated metabolites during seed development in two soybean cultivars with different protein and oil contents. KEGG enrichment analysis indicated that the enriched pathways included plant hormone signal transduction, phenylpropanoid biosynthesis, linoleic acid metabolism, glycerolipid metabolism, carbon metabolism, and the biosynthesis of amino acids and secondary metabolites. The authors proposed that soybean varieties with high seed protein contents are prone to delayed leaf senescence as well as earlier photomorphogenesis.

Raffinose family oligosaccharides (RFOs) are abundant in seeds and are difficult for humans and animals to digest, causing flatulence and severe abdominal discomfort. Most leguminous seeds, such as chickpea (*Cicer arietinum*), have noticeable amounts of RFOs. Elango et al. used high-performance liquid chromatography to measure the contents of RFOs (raffinose and stachyose), ciceritol, and sucrose in chickpea and identified genes involved in sugar metabolism and transport via genome-wide association mapping. Accessions having lower RFOs and higher sucrose concentrations might be desirable for use in breeding.

Much is known about the genetic factors regulating isoflavone concentrations in soybean seeds; these bioactive compounds are important for both plants and humans. Chen et al. analyzed the dynamic variation in isoflavone contents and the isoflavone accumulation patterns in soybean at the physiological level using eight RILs. They also investigated the whole-genome expression profiles of four lines as well as 42 meta-transcriptome datasets and identified molecular modules strongly associated with isoflavone concentration and genes affecting isoflavone accumulation in developing seeds. Their findings increase our understanding of the biosynthesis and molecular control of isoflavones at the physiological and molecular levels and will assist in breeding new soybean cultivars having higher isoflavone concentrations.

## 4 Abiotic stress resistance

Like soybean seeds, the seeds of the legume crop peanut, or groundnut (*Arachis hypogaea*), are rich in protein and oil. Zhang X et al. characterized the aldehyde dehydrogenase (ALDH, EC: 1.2.1.3) family in *A. hypogaea*. Of the 71 members of the peanut ALDH superfamily (*AhALDH*), some genes exhibited tissue-specific expression, and some were differentially expressed upon exposure to saline-alkali stress, implying their possible involvement in plant responses to abiotic stress. These findings can be utilized to breed peanut with improved stress resistance.

Cytokinin oxidase/dehydrogenases (CKXs) regulate plant growth and development by irreversibly degrading cytokinin, thus helping plants cope with environmental stress. Du et al. characterized the GmCKX gene family in soybean using transcriptome profiling (RNA-seq), qRT-PCR, and bio-informatics tools. Eighteen *GmCKX* genes were identified in soybean and clustered into five clades, some of which exhibited tissue-specific expression. RNA-seq analysis demonstrated that GmCKXs may have crucial functions in plant responses to salt and drought stress. Abiotic stress reduced zeatin contents in soybean radicles but increased CKX activity. External application of the phytohormones 6-benzylaminopurine (6-BA) and indole-3-acetic acid (IAA; auxin) promoted transcriptional abundance of *GmCKX10* and *GmCKX18* but hampered the expression of *GmCKX1*, *GmCKX6*, and *GmCKX9*, reducing the zeatin contents in radicles.

Basic leucine zipper domain (bZIP)–containing transcription factors are key factors in regulating environmental signaling and stress responses, as well as carbon–nitrogen balance. Yue et al. reviewed the roles of 161 bZIP family members in soybean, which could be classified into 13 groups. Physiological analyses and genetic engineering have revealed the functions of several soybean bZIP transcription factors. However, the biochemistry and regulatory mechanisms of most bZIP transcription factors in soybean remain unclear.

## 5 Nodulation

Symbiotic nitrogen fixation is an important component of the nitrogen cycle. Enhancing this process in legumes could facilitate sustainable agriculture, as symbiotic nitrogen fixation contributes almost 20% of the nitrogen needed for global grain and oilseed production.

Nodulation is controlled via an exquisite network consisting of various nodulation-related genes. Zhang Y et al. characterized the function of the TOC1 family member GmTOC1b during nodulation in soybean. Mutants of *GmTOC1b* displayed more numerous and heavier nodules than the wild type due to increased root hair curling and infection thread production, whereas overexpressing *GmTOC1b* inhibited nodulation. Furthermore, the authors determined that GmTOC1b binds to the promoters of nodulation-related genes such as *GmNIN2a* and *GmENOD40-1* to negatively regulate their transcriptional abundance. These findings reveal the crucial function of GmTOC1b in controlling nodule formation in soybean.

The studies highlighted here illuminate the genetic basis of yield- and quality-related traits in legumes, potentially spurring new research on these exciting topics and facilitating the genetic improvement of many important crops.

## Author contributions

ZX: Writing – original draft. QS: Writing – review & editing. KH: Writing – review & editing. JC: Writing – review & editing. CZ: Writing – review & editing.
